# Outcome of hearing preservation related to tumor morphologic analysis in acoustic neuromas treated by gamma knife radiosurgery

**DOI:** 10.1186/s13014-017-0875-z

**Published:** 2017-08-15

**Authors:** Szu-Yen Pan, Shih-An Liu, Ming-Hsi Sun, Hsi-Kai Tsou, Shinh-Dung Lee, Yen-Ju Chen, Jason Sheehan, Meei-Ling Sheu, Hung-Chuan Pan

**Affiliations:** 1 Department of Neurosurgery, Taichung Veterans General Hospital, 1650 Taiwan Boulevard Sec.4, 40705 Taichung, Taiwan; 2 Faculty of Medicine, School of Medicine, National Yang-Ming University, Taipei, Taiwan; 3 Department of Neurosurgery, University of Virginia, Charlottesville, VA USA; 4 Institute of Biomedical Science, National Chung-Hsin University, Taichung, Taiwan

**Keywords:** Acoustic neuroma, Gamma knife radiosurgery, Tumor morphology

## Abstract

**Background:**

Gamma Knife radiosurgery (GKRS) is an important part of the neurosurgical armamentarium in the treatment of acoustic neuromas. However, the treatment outcome related to the morphology of the tumor has not been rigorously studied. In this cohort, we evaluated the morphological features of the tumor in the tumor response and neurological outcomes after GKRS.

**Material and methods:**

From July 2003 to December 2008, there were 93 cases of acoustic neuromas treated upfront with GKRS with 64 cases with serviceable hearing and 29 cases without serviceable hearing to fulfill the margin dose of 12Gy with at least follow up 5 years.

**Results:**

The duration of symptom before GKRS in serviceable /no serviceable hearing was 7.9 ± 1.2 and 15.3 ± 3.1 months (*p* < 0.001) and associated no-hearing symptom was 70% and 35%, respectively (*p* < 0.001). There was 81.2% of hearing preservation after GKRS in serviceable hearing group including 27 cases of pear type (84%), 14 of linear type (70%), and 9 cases of sphere type (90%) (*p* < 0.01); however, there was no case of hearing improvement in the no-serviceable hearing group (0 of 29). There were 85% of patients with decreased tinnitus in serviceable hearing groups as compared to 61.5% of patients in no serviceable hearing group (*p* < 0.05). In multivariate analysis, the tumor morphology was highly correlated to hearing preservation rate (*p* < 0.01).

**Conclusion:**

In the limited case of this cohort, we found that the tumor morphology and timing of treatment was highly correlated to the rate of hearing preservation. The sphere type of tumor morphology was associated with the best chance of hearing preservation.

## Introduction

Gamma Knife Radiosurgery (GKRS) serves as a powerful tool in the neurosurgical armamentarium for the treatment of small to moderately sized acoustic neuromas [1, 2]. In large acoustic tumor, hearing and facial preservation in groups treated with subtotal removal by microsurgery followed by radiosurgery seem better those treated by microsurgery alone [3–5]. In small to moderately sized tumors treated by microsurgery, the facial nerve preservation rate generally was more than 90%, but the hearing preservation was as low as 20% even in the hands of experienced surgeons [6–8]. The hearing preservation in microsurgery was highly correlated with the size of tumor with hearing preservation in one series being 52% in tumors less than 2 cm and 83% in tumors less than 1 cm [9]. In a recent GKRS series with peripheral doses between 11 and 13 Gy, the hearing preservation rate ranged from 41 to 78% and facial nerve preservation was nearly 100% [10–16]. Concerning the treatment outcome of facial and hearing preservations, GKRS seem a reasonable and the preferred choice for patients harboring small to medium size of acoustic neuromas.

There were several factors contributing to high hearing preservation rate including the peripheral dosage of tumor, tumor size, cochlear dose, volume of intracanalicular portion of the tumor, and pre-radiosurgical hearing capacity [15–20]**.** In general, a margin dosage less than 13 Gy were more likely to preserve hearing compared to earlier series that used higher margin doses [17]**.** With dose less than 12.5Gy, further reduction in cranial neuropathy was observed [21]**.** Either volume of tumor or tumor growth rate remain controversial in predicting the preservation of hearing after GKRS and some have even implicated that a large tumor did not increase the risk of hearing loss as compared to the smaller tumor [17, 22]**.** Those with pre radiosurgical hearing function of Gardner-Robertson Class I showed better preservation than those GRC of II-IV [10, 14]**.** The cochlear dosage limited within 4 to 5.3 Gy showed the better hearing preservation and further reduction to a dose less than 2.7 Gy exerted still better hearing preservation [12, 13, 15, 23]**.** In addition, the subset of tumor morphology with large intracanaicular volume exerted worse hearing preservation [16, 24, 25].

As known, the tumor volume alteration responded to gamma knife treatment was different with smaller volume change in the intracranial portion and larger tumor response outside the intracranial portion [26]**.** In addition, the scope of brain stem compression related to hearing preservation was still debated [10, 27]**.** The shape and morphologic alteration of acoustic neuromas subjected to gamma knife treatment and their implications in terms of hearing preservation have not been rigorously investigated. In this study, we retrieved the data from our radiosurgical patient registry and investigated the relationship of the shape of tumor, grade of brainstem compression, pre-radiosurgical hearing function and associated symptoms, and radiosurgical parameters to radiological tumor response and hearing preservation after GKRS.

## Material and methods

### Patient population

From July 2003 to December 2008, there were 150 cases of acoustic schwannoma treated with GKRS. There were 32 cases with previous operation, 10 with neurofibromatosis type II, and 10 cases without regular follow up which were excluded in this study. Seven cases were excluded from this study due to not be categorized into either of three types of tumor morphology. Finally, there were only 93 cases entered into this study. The data analysis has been approved by Taichung Veterans General Hospital Institute Review Board (CG12319B).

### Gamma knife surgical dose planning

Stereotactic radiosurgery was performed using a Leksell Gamma Knife model 4C (Elekta AB). Treatment planning was performed using Leksell GammaPlan software (version 5.3, Elekta AB). A dose of 12 Gy was prescribed to the 50% isodose line, and it covered more than 95% of the tumor in all treatments.

### Clinical follow-up

Patients were followed up at our otolaryngology and neurosurgical outpatient clinics 3 months after radiosurgery and then 6 months periodically until the last follow up. MRI follow up was conducted 6 months after GKRS and then yearly until last follow up. The clinical data obtained at follow-up included the audiogram and a detailed neurological function assessment.

### Assessment of hearing function

Serviceable hearing was defined as a speech repetition threshold or pure tone audiogram value less than 50 dB and a speech discrimination score of 50% or greater.

### House-Brackmann facial grading system

The HB scale was used to approximate the quantity of facial nerve function that the patient had at presentation as well as at each follow-up interval. The HB scale has 6 grades and each grade is reported as a fraction (for example, 1/6 = Grade I). In the HB scale, Grade I indicates perfectly normal, Grade II indicates slight or mild weakness, Grade III indicates moderate weakness with good (normal) eye closure, Grade IV indicates moderate weakness with no volitional eye closure, Grade V indicates severe weakness, and Grade VI indicates total facial paralysis.

### Definition of tumor morphology

The MR imaging slice thickness was 1-2 mm for T1-weighted images or time of flight with and without Gd contrast administration for tumor morphologic assessment. The MR imaging slice thickness was 1-2 mm for T2-weighted images and FLAIR without contrast administration for the assessment of surrounding tissue reaction. The tumor volumes were determined on MR imaging images using a picture archiving and communications system (PACS) and GKRS planning for further assessment.

**Fig. 1:**
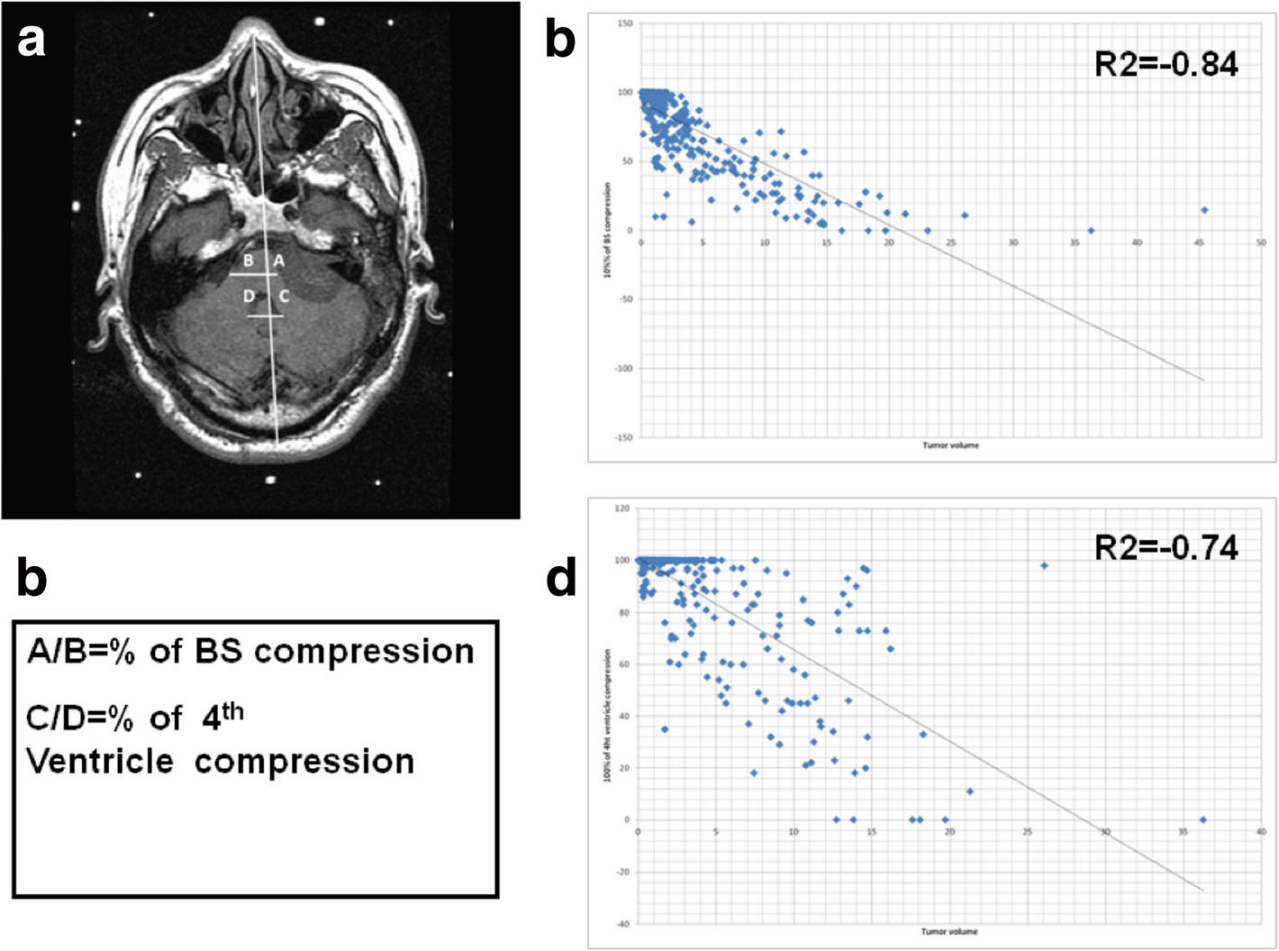
Illustration of calculation of ratio of brain stem and fourth ventricle compression and related to tumor volume (**a**) Illustration of ratio of brain stem and 4th ventricle compression in T1 weight without contrast of MRI (**b**) The formula for definition of ratio of compression (**c**) Plot of brain stem compression related to tumor volume (**d**) Plot of 4th ventricle compression related to tumor volume

The calculation of brainstem compression was assessed including the brainstem compression and fourth ventricle compression ratio. For this calculation, the A line was determined from the midpoint between bilateral internal carotid artery and junction of bilateral transverse sinus. B line was drawn perpendicular to the A line at the maximum site of tumor compression. The distance of the tumor to the A line divided by the distance from the brainstem surface to the A line was determined as brain stem compression. C line was determined by the line perpendicular to A line at the maximum diameter of 4th ventricle. The distance of border of the fourth ventricle to A line divided by the distance from the fourth ventricle surface to A line at the opposite side was determined as the degree of fourth ventricle compression (Fig. 1).

**Fig. 2:**
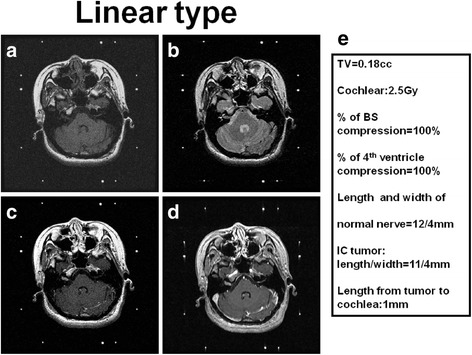
Illustration and definition of tumor morphology of linear type (**a**) T1 weighted MRI imaging of right acoustic tumor (**b**) T2 weighted MRI imaging of right acoustic tumor (**c**) T1 weighted with contrast administration of MRI imaging of right acoustic tumor (**d**) Time of flight with contrast administration of MRI imaging of right acoustic tumor (**e**) Representative of data analysis in this case

**Fig. 3:**
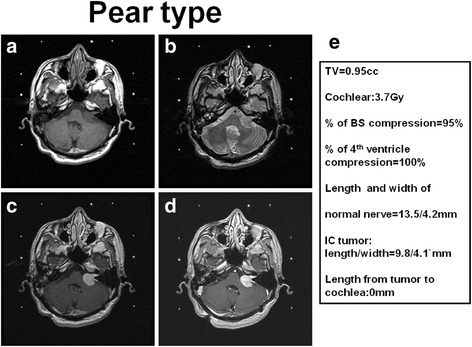
Illustration and definition of tumor morphology of pear type (**a**) T1 weighted MRI imaging of right acoustic tumor (**b**) T2 weighted MRI imaging of right acoustic tumor (**c**) T1 weighted with contrast administration of MRI imaging of right acoustic tumor (**d**) Time of flight with contrast administration of MRI imaging of right acoustic tumor (**e**) Representative of data analysis in this case

**Fig. 4:**
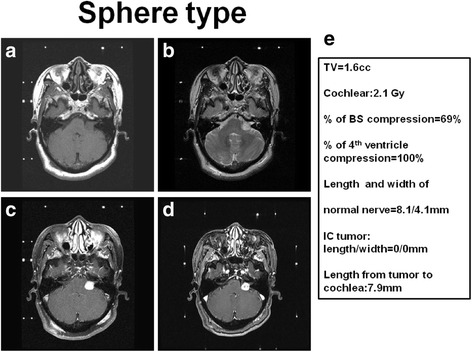
Illustration and definition of tumor morphology of sphere type (**a**) T1 weighted MRI imaging of right acoustic tumor (**b**) T2 weighted MRI imaging of right acoustic tumor (**c**) T1 weighted with contrast administration of MRI imaging of right acoustic tumor (**d**) Time of flight with contrast administration of MRI imaging of right acoustic tumor (**e**) Representative of data analysis in this case

A line to determine the intra/extracanalicular tumor /nerve was made along the ridge of petrous bone to posterior surface of the petrous bone. The tumor lateral to this line was considered to intracanalicular part and medial to this line was considered to extracanalicular part. The total length of facial-vestibular bundle was determined from the surface of brainstem to cochlea. The nerve width was determined by the maximum width of the nerve bundle in the consecutive MRI. The tumor morphology was categorized into three types. The linear type was defined as the morphology of tumor when it was linear shaped either with the internal canal, outside the canal, or combined both (Fig. 2). The pear type was defined as when the majority of the tumor was sphere with a stalk projected into the intracanalicular portion (Fig. 3). The sphere type was defined as the tumor morphology of sphere without any part of tumor projected into intracanalicular portion (Fig. 4).

### Statistical analysis

The descriptive statics were computed using the standard methods to calculate median and mean values. Univariate and multivariate analysis were performed to assess for variable predictive of preservation of serviceable hearing after GKRS. The following variables were assessed: the tumor morphology, length of tumor in IAC, width of tumor in IAC, percentage of brain stem compression, percentage of fourth ventricle compression, cochlear dosage, dosage to the lateral semicircular canal, dosage to the median semicircular canal, dosage to the inferior semicircular canal, maximum brainstem (BS) dosage, and 10 Gy volume in brainstem. The unpaired student t test or analysis of variance was used for continuous variables. Nominal or ordinal data were compared using the two-tailed Fisher exact test. All statistical analyses were conducted with the aid of commercially available software (SPSS 16.0). Statistical significance was defined as a probability of value less than or equal to 0.05.

## Results

### Patient, tumor, and treatment parameters

There were 93 cases in which GKRS was used as the upfront treatment. The male to female ratio was 60 to 33. The right and left lateralization was 60 to 33. The median age was 58(57.7 ± 15.1) years old. The follow up period was 76.3 ± 3.6 months. There were 64 cases with serviceable hearing and 29 cases without serviceable hearing at the time of presentation for GKRS. There were 5 cases that presented with hemifacial spasm. One case presented with grade I facial palsy. There were 46 cases with tinnitus and 7 cases of facial numbness. One case had trigeminal neuralgia. In imaging analysis, there were 20 cases with tumor invasion to trigeminal nerve. There were 11 cases of brainstem dysfunction. There were 43 cases of tumor involved the whole vestibular-facial nerve bundles and 50 cases with only part of the tumor located within the IAC. The mean tumor volume was 3.14 ± 0.05 cm^3^. The length of opposite side of vestibular-facial nerve bundle was 19.9 ± 0.2 mm. The mean diameter of opposite side of ICA was 4.3 ± 0.6 mm. The morphology analysis showed 60 cases with pear type, 22 cases of linear type, and 11 cases of sphere type.

The demography of patient without operation before GK was sub-categorized into serviceable hearing and no serviceable hearing showed in Table 1. There was no significant difference between groups in patients’ age, sex, tumor lateralization, duration of symptoms, clinical symptoms, tumor morphology, mathematic analysis of cochlear nerve length, tumor length and width in IAC, and radiation dosage. However there was significant difference in the interval from the perception to symptom and treatment (7.9 ± 1.2 months in serviceable hearing versus 15.3 ± 3.1 months in no serviceable hearing) (*P* < 0.05)

**Table 1:** Demography of patients in serviceable hearing and no serviceable hearing

	Serviceable hearing	No Serviceable hearing
n	64	29
Median age	54	63
Follow up(months)	77.9 ± 4.5	72.6 ± 5.9
M/F	38/26	22/7
Rt/Lt	34/30	15/14
Duration of S/S	7.9 ± 1.2	15.3 ± 3.1
Facial spasm	3	2
tinnitus	33	13
Trigeminal dysfunction	8(1 TN)	1
Brain stem dysfunction	8	1
Tumor type	Linear = 21, pear = 34, sphere = 9	Linear = 5, pear = 19, sphere = 5
Trigeminal N compression	16	10

The data presented with mean ± standard errors

The anatomic structure and associated radiation dosage are depicted in Table 2. There was no significant difference between groups in tumor volume, percentage of brainstem compression, percentage of fourth ventricular compression, peripheral dose and associated peripheral dose line, dose to the cochlea, semicircular canal, and brainstem maximum doses. There was significant difference in length of nerve to cochlea (0.3 ± 0.04 mm versus 0.25 ± 0.07, *p* < 0.05), length (0.66 ± 0.04 versus 0.77 ± 0.04 mm, *p* < 0.05) and width of tumor (0.46 ± 0.03 mm versus 0.55 ± 0.04 mm, *p* < 0.05) in ICA and 10 Gy volume in brain stem (0.13 ± 0.03 cm^3^ versus 0.15 ± 0.06 cm^3^, *p* < 0.05).

**Table 2:** Anatomic structure and associated surrounding radiation dose in serviceable hearing and no serviceable hearing

	Serviceable hearing	No Serviceable hearing
Length of nerve to cochlea(mm)	0.3 ± 0.04	0.25 ± 0.07
Tumor volume (CC)	2.9 ± 0.5	3.7 ± 1.3
Length of tumor in IAC (mm)	0.66 ± 0.04	0.77 ± 0.04
Width of tumor in IAC (mm)	0.46 ± 0.03	0.55 ± 0.04
Percentage of BS compression	0.8 ± 0.03	0.8 ± 0.06
Percentage of 4th ventricle compression	0.89 ± 0.02	0.86 ± 0.06
Peripheral treatment dosage (Gy)	12 ± 0.05	12.1 ± 0.08
Cochlea dosage (Gy)	3.3 ± 0.4	3.5 ± 0.6
Lateral semicircular canal dosage (Gy)	2.73 ± 0.24	2.98 ± 0.42
Medial semicircular canal dosage (Gy)	2.89 ± 0.21	3.26 ± 0.41
Inferior semicircular canal dosage (Gy)	3.33 ± 0.24	3.14 ± 0.31
BS dosage (Gy)	8.7 ± 0.5	7.9 ± 0.1
10 Gy volume in BS (cc)	0.13 ± 0.03	0.15 ± 0.06
Percentage in peripheral (%)	51.1 ± 0.6%	50.8 ± 0.7%

The data presented with mean ± standard errors

### Outcomes after stereotactic Radiosurgery

**Fig. 5:**
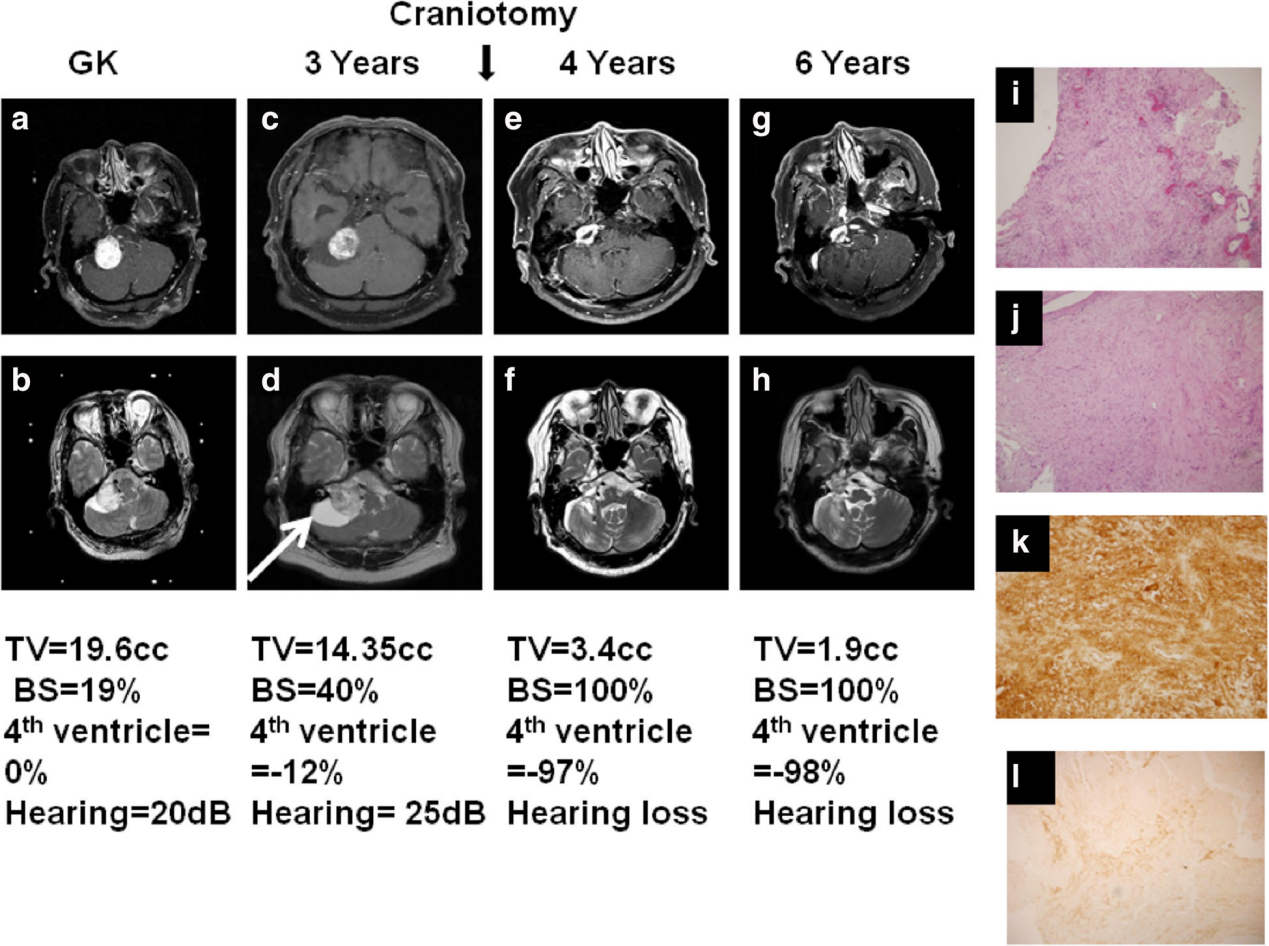
Illustration of sphere type of right acoustic tumor subjected to tumor resection due to no-hearing associated symptom (**a**) T1 weighted MRI imaging with contrast administration at time of GK (**b**) T2 weighted MRI imaging at time of GK (**c**)T1 weighted MRI imaging with contrast administration 3 years after GK (**d**) T2 weighted MRI imaging 3 years after GK (**e**)T1 weighted MRI imaging with contrast administration 4 years after GK (**f**) T2 weighted MRI imaging 4 years after GK (**g**)T1 weighted MRI imaging with contrast administration 6 years after GK (**h**) T2 weighted MRI imaging 6 years after GK (**i**) H& E staining in central part of tumor scattered with hemorrhage (×40) (**j**) H&E staining in peripheral part of tumor(×100) (**k**) Immunohistochemistry staining of S100 of tumor(×100) (**l**) Immunohistochemistry staining of EMA(×100). Back arrow indicated the time point of craniotomy for tumor removal. White arrow indicated the peri-tumor arachnoid cyst

**Fig. 6:**
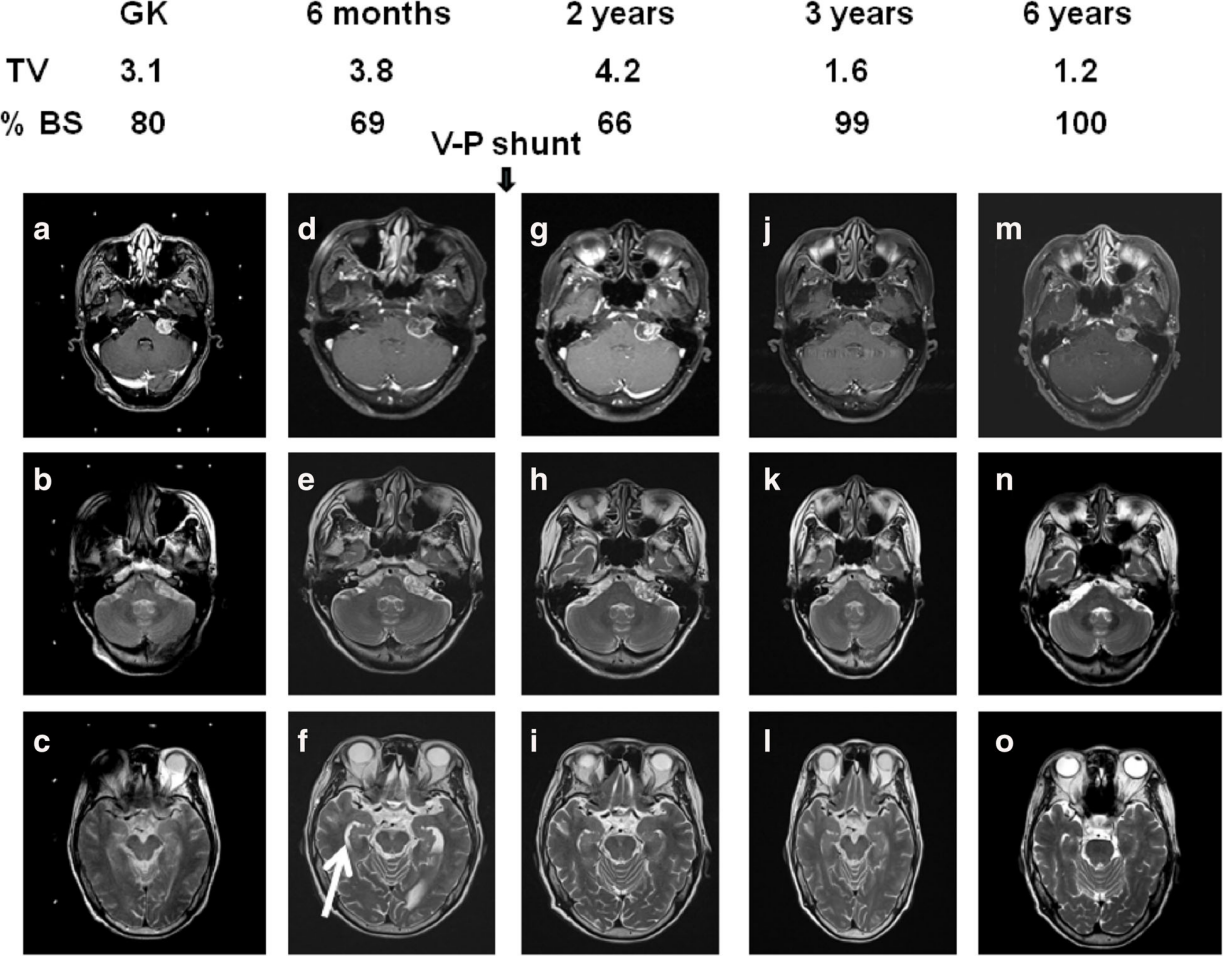
Illustration of pear type of acoustic tumor subjected to gamma knife treatment with hydrocephalus (**a**) T1 weighted MRI imaging with contrast administration at time of GK (**b**) T2 weighted MRI imaging at time of GK(**c**) T2 weighted MRI imaging at time of GK for assessment of size of temporal horn (**d**) T1 weighted MRI imaging with contrast administration 6 months after GK (**e**) T2 weighted MRI imaging 6 months after GK (**f**) T2 weighted MRI imaging 6 months after GK for assessment of size of temporal horn (**g**) T1 weighted MRI imaging with contrast administration 2 years after GK (**h**) T2 weighted MRI imaging 2 years after GK(**i**) T2 weighted MRI imaging 2 years after GK for assessment of size of temporal horn (**j**) T1 weighted MRI imaging with contrast administration 3 years after GK (**k**) T2 weighted MRI imaging 3 years after GK (**l**) T2 weighted MRI imaging 3 years after GK for assessment of size of temporal horn (**m**) T1 weighted MRI imaging with contrast administration 6 years after GK (**m**) T2 weighted MRI imaging 6 years after GK(**o**) T2 weighted MRI imaging 6 years after GK for assessment of size of temporal horn. White arrow indicated the region of temporal horn

The outcomes of GKRS in terms of hearing and associated symptom and radiologic response are detailed in Table 3. There was 81.2% of hearing preservation in serviceable hearing group. However, there were only 34.5% of cases with stable hearing in non-serviceable hearing group (*P* < 0.01). There were 85% of patients with decreased tinnitus after GKRS, and 61.5% of patients in non-serviceable hearing group had decreased tinnitus (*p* < 0.05). One of 64 cases in the serviceable hearing presented with severe dizziness and underwent a tumor resection with improvement of dizziness but resulting hearing loss (Fig. 5). There was one case in which the patient developed hydrocephalus and underwent a ventriculoperitoneal shunt due to an unsteady gait. The patient obtained an excellent clinical outcome after operation (Fig. 6).

**Table 3:** Outcome of hearing preservation and associated symptom allocated to serviceable hearing and no serviceable hearing

	Serviceable hearing	No Serviceable hearing
Patient number	64	29
Hearing loss	2(3%)	10(34.5%)
Hearing stable	52(81.2%)	11(38%)
Hearing decrease	9(14%)	8(27.5%)
Hemifacial spasm	2/3(improved)	2/2 decreased
Tinnitus	28/33(decreased)85%	8/13(improved) 61.5%
Facial palsy	0	0
Increased size of tumor	1(craniotomy)	1(V-P shunt)

### Tumor morphology and outcome

The morphological analysis of tumor related to hearing outcome was assessed below. In the serviceable hearing group, the morphology of sphere type showed 90% of hearing preservation (9 of 10 patients) as compared to pear type (84% hearing preservation) (27 of 32 patients) and linear type (70%) (14 of 20 patients). In the non-serviceable hearing group, there was 48% of linear group (11 of 23 patients) and 40% of linear group (2 of 5 patients) retained stable hearing. However, there was no patient who obtained serviceable hearing after GKRS in the non-serviceable hearing group.

The distributions of patients by tumor morphology is illustrated in Table 4. There was a high incidence of none hearing associated symptom including of tinnitus, trigeminal neuralgia and hemifacial spasm in the pear type tumor morphology. The hearing outcome subjected to morphology analysis in Table 5 showed that a larger volume was distributed in the sphere type followed by the pear and linear types (*P* < 0.01). The length of tumor in the IAC were significant higher in the linear type than the pear and sphere type (*p* < 0.01). The width of tumor in the IAC was significant higher in the linear and pear type than in sphere type (*p* < 0.01). The percentage of brainstem compression was higher in the sphere and pear types than in linear type (*p* < 0.05). The percentage of 4th ventricle compression was significant higher in sphere type than the pear and linear types (*p* < 0.01).There was significant higher cochlea dose in the linear and pear type than the sphere type (*p* < 0.01). The dose to the semicircular canal was higher in the linear and pear types than in sphere type (*p* < 0.01; *p* < 0.01; *p* < 0.01). There were significant higher dosage to the brainstem in the sphere and pear type than linear type (*p* < 0.01). The associated brainstem 10 Gy volume was also higher in the sphere and pear type than the linear type.

**Table 4:** Demography in serviceable hearing group allocated by tumor morphology

	Linear	Pear	Sphere9
Number	14	27	9
Median age	55	53	56
Follow up(months)	76.3 ± 5.9	73 ± 4.8	71 ± 7.1
M/F	8/6	16/11	6/3
Rt/Lt	8/6	14/13	5/4
Duration of S/S	7.1 ± 1.3	8.1 ± 2.1	7.8 ± 1.5
Facial spasm	0	2	1
tinnitus	9(64.3%)	23(85.1%)	1(11.1%)
Trigeminal dysfunction	1	6	2
Brain stem dysfunction	0	6	2
Trigeminal N compression	0	12	4

The data presented with mean ± standard errors

**Table 5:** Factors analysis related to hearing preservation allocated by tumor morphology in serviceable hearing patients

	Linear	Pear	Sphere	*P* value
Tumor volume(cc)	0.82 ± 0.03	2.8 ± 0.3	3.1 ± 0.2	*P* < 0.001
Length of tumor in IAC(mm)	0.71 ± 0.03	0.43 ± 0.03	0.05 ± 0.03	*P* < 0.01
Width of tumor in IAC(mm)	0.48 ± 0.05	0.44 ± 0.03	0.1 ± 0.05	*P* < 0.01
% of BS compression	0.98 ± 0.04	0.75 ± 0.06	0.71 ± 0.04	*P* < 0.05
% of 4th ventricle compression	0.96 ± 0.07	0.81 ± 0.03	0.67 ± 0.04	*P* < 0.01
Peripheral dosage (Gy)	12	12	12	NA
Cochlea (Gy)	4.1 ± 0.3	3.6 ± 0.3	1.2 ± 0.2	*P* < 0.01
Lateral semicircular canal (Gy)	2.8 ± 0.2	2.7 ± 0.3	1.1 ± 0.1	*P* < 0.01
Medial semicircular canal (Gy)	2.7 ± 0.2	2.6 ± 0.2	1.2 ± 0.1	*P* < 0.01
Inferior semicircular canal (Gy)	2.8 ± 0.3	2.9 ± 0.5	1.2 ± 0.2	*P* < 0.01
BS dosage (Gy)	2.3 ± 0.1	9.1 ± 0.2	8.6 ± 0.3	*P* < 0.01
10 Gy volume in BS (Gy)	0.01 ± 0.005	0.17 ± 0.04	0.23 ± 0.03	*P* < 0.01
Percentage in peripheral (%)	50	50	50	NA

The data presented with mean ± standard errors

The uni-variate and multi-variate analyses are shown in Table 6. In the uni-variate analysis, the rate of hearing preservation was highly correlated to the tumor morphology, length of tumor in IAC, width of tumor in IAC, percentage of brainstem compression, percentage of 4th ventricle compression, cochlear dosage, dose to the lateral semicircular canal, dose to the median semicircular canal, dose to the inferior semicircular canal, BS dose, and 10 Gy volume in the brainstem. In multivariate analysis, only the tumor morphology demonstrated significantly prognostic importance with regard to hearing preservation at last follow up.

**Table 6:** Univariate and multivariate analysis in hearing preservation in patient with serviceable hearing after GKRS

	Univariate		Multivariate		95% CI
	*P*	HR	*p*	HR	
Tumor morphology	*P* < 0.01	4.1	*P* < 0.01	4.45	2.1-7.9
Length of tumor in IAC	*P* < 0.01	3.68	*P* = 0.15	1.92	0.9-3.1
Width of tumor in IAC	*P* < 0.05	2.93	*P* = 0.23	2.1	1.1-2.9
Percentage of BS compression	*P* < 0.05	3.12	*P* = 0.22	1.77	1.3-3.1
Percentage of 4th ventricle compression	*P* < 0.05	3.42	*P* = 0.19	1.67	0.7-3.1
Cochlear dosage	*P* < 0.01	4.2	*P* = 0.09	2.11	0.8-3.2
Lateral semicircular canal dosage	*P* < 0.05	3.51	*P* = 0.35	1.88	1.1-3.1
Medial semicircular canal dosage	*P* < 0.01	4.33	*P* = 0.21	1.62	0.7-3.1
Inferior semicircular canal dosage	*P* < 0.01	3.77	*P* = 0.19	1.57	0.8-2.7
BS dosage	*P* < 0.05	2.95	*P* = 0.39	1.44	0.6-2.8
10 Gy volume in BS	*P* < 0.05	3.15	*P* = 0.52	1.39	0.4-2.2

*P p* value

*HR* hazard ratio

*95% CI* confidence interval

## Discussion

Beyond local tumor control and maintenance of facial nerve function, hearing preservation is a paramount outcome to assess after GKRS. The initial tumor volume was a crucial factor to determine the radiologic response after GKRS. However, Gamma Knife treatment parameters can be correlated to tumor volume including the radiation dosage to the cochlea, brainstem, and semicircular canal [17, 22]**.** In this study, the tumor volume seemed to be larger in the sphere type, but this same morphological type predicted improved high hearing preservation despite the overall larger tumor volume. In contrast, the morphology of the tumor rather than tumor volume better predicted the probability of hearing preservation.

The location of tumor and degree of brainstem compression were reasonable predictors of hearing outcome after GKRS. There was higher hearing preservation in tumor without the involvement of the whole internal acoustic canal, a greater distance away from the cochlea, and less brainstem compression [16–20, 22, 23]**.** In this study, we found that the morphology of the sphere type demonstrated better hearing preservation rate as compared to the pear and linear type of tumors. The ratio of brain stem compression in sphere type was highly less than the pear type but there was no significant difference between the pear types. This data suggests that brainstem compression was not a key factor to predicting the outcome of hearing preservation. On the contrary, the initial tumor growth pattern was predictive of hearing preservation at last follow up.

The timing of treatment for acoustic neuromas especially in those patients with small-medium size of tumor is hotly debated. There were some reports concerning the treatment of acoustic tumor with deterioration of hearing after GKRS and considered to be an adverse effect from the radiation. “Wait and see” treatment option is advocated in some studies. But there were also researchers that encourage the treatment as soon as possible and against the “wait and see” policy [26, 28, 29]**.** In this study, we found that a high proportion of serviceable hearing was noted in those patients with shorter interval of symptom as compared to those longer symptoms. In addition, there was no patient without serviceable hearing that regained serviceable hearing after GKRS. These data support early treatment as a crucial factor for better hearing preservation.

The response of no hearing associated factors after GKRS seemed to be a facilitated factor for assessment of hearing outcome. In some reports, the regression of no hearing associated factor exerted the higher hearing preservation rate than those without regression [8, 30]**.** This study found that no hearing associated symptom was significantly higher in serviceable hearing than in the serviceable hearing group, but the higher regression of no hearing associated symptom was noted in the serviceable hearing. It can be explained to be that the no hearing associated symptom appeared earlier than hearing impairment symptom and it forward the patient to receive examination and entered into the treatment program.

In this study, most of the small to medium sized acoustic neuromas can be morphologically categorized into the sphere, pear and linear types. Based on the classification, the sphere type of tumor had the original site of tumor growth outside the internal acoustic canal; the pear type of tumor had the growth pattern either from the originality outside the IAC with some part of tumor invasive to proximal part of IAC or from the distal part of IAC with most of tumor invasive outside the IAC; the pear type had the originality of tumor in the ICA canal. There was a high degree of hearing preservation in the sphere type followed by the pear and to a lesser extent the linear type. This suggests that of the tumor growth and morphology seem to be a crucial determinant in predicting the result of hearing outcome after GKRS.

There were several limitations in this study. First, some of acoustic neuroma could not be subcatergozied into either of linear, pear and sphere type and it reduced the power in prediction of hearing preservation. The numbers of slices obtained from MRI for the calculation of values in anatomic structure were various in different sizes of tumor (that is, the larger slice number in bigger tumor), which may be a confounding factors in measurement [31]**.** The number of cases in this series is small, which attenuated the statistic power in prediction.

## Conclusion

Those with fewer symptoms and favorable hearing status at GKRS were more likely to have hearing function at the last follow up. The tumor morphology influenced the outcome of hearing and those with a spherical tumor were likely to have high hearing preservation rate.

## Abbreviations

GKRS: Gamma Knife radiosurgery
HB: House-Brackman
IAC: Internal acoustic canal
MRI: Magnetic resonance imaging
PACS: Picture archiving and communications system
